# Annual Acknowledgement of Reviewers

**DOI:** 10.1186/s12860-016-0082-z

**Published:** 2016-02-12

**Authors:** Timothy R. Sands

**Affiliations:** BioMed Central, Floor 6, 236 Gray’s Inn Road, London, WC1X 8HB, UK

## Abstract

The editors of BMC Cell Biology would like to thank all those who have contributed reviews to the journal in Volume 16 (2015).

**Nivea Amoêdo**

Brazil

**Michelle Barton**

USA

**Ilse Beck**

Belgium

**Claudio Brancolini**

Italy

**Anton Burakov**

Russia

**Matthew Butchbach**

USA

**Rachel Chan**

UK

**Yang Chen**

USA

**Frank Claessens**

Belgium

**Matthias Falk**

USA

**So-Ichiro Fukada**

Japan

**Yasuhisa Fukui**

Taiwan

**Noor Gammoh**

UK

**Ian Ganley**

UK

**Minghui Gao**

USA

**Sudhiranjan Gupta**

USA

**Vsevolod Gurevich**

USA

**Hyunjung Ha**

South Korea

**John Haley**

USA

**Troy Harkness**

Canada

**Naohiro Hashimoto**

Japan

**Sudan He**

China

**James Hudson**

Australia

**Eihachiro Kawase**

Japan

**Conor Kearney**

Ireland

**Leung Kim**

USA

**Jae Kim**

South Korea

**Takao Kinjo**

Japan

**Anna Kudryavtseva**

Russia

**Li Lan**

USA

**Julie Lefebvre**

Canada

**Anne Lenferink**

Canada

**Steve Ley**

UK

**Xing Liu**

China

**Andreas Ludwig**

Germany

**Xianghang Luo**

China

**Patricia Martin**

UK

**Agustin Martinez**

Chile

**Tetsuo Maruyama**

Japan

**Dennis McCance**

UK

**Lindolfo Meirelles**

Brazil

**Andreas Merdes**

France

**Takayuki Morisaki**

Japan

**Ciaran Morrison**

Ireland

**Yosuke Nagata**

Japan

**Masashi Naito**

Japan

**Meera Nanjundan**

USA

**Tsutomu Nohno**

Japan

**Dulal Panda**

India

**Imara Perera**

USA

**Michael Ristow**

Switzerland

**Adolfo Saiardi**

UK

**Juliane Salbach-Hirsch**

Germany

**Puran Sijwali**

India

**Anurag Singh**

USA

**Andrew Smith**

UK

**Daniel Sun**

USA

**Bong Hwan Sung**

USA

**Hiroyuki Tamemoto**

Japan

**Raija Tammi**

Finland

**Satoshi Tashiro**

Japan

**Andrea Tomasella**

Italy

**Silvia Tyciakova**

Slovakia

**Madoka Uezumi**

Japan

**Francesc Ventura Pujol**

Spain

**Daniele Veritti**

Italy

**Davide Vigetti**

Italy

**Samuel Wilson**

USA

**Anthony Wright**

Sweden

**Bin Xiao**

China

**Ding Xie**

China

**Akira Yasui**

Japan

**Jie Zheng**

China

